# Artifact removal in the contour areas of SAXS-CT images by Tikhonov-L1 minimization

**DOI:** 10.1107/S1600576721011523

**Published:** 2021-11-30

**Authors:** Hiroki Ogawa, Shunsuke Ono, Yuki Watanabe, Yukihiro Nishikawa, Shotaro Nishitsuji, Taizo Kabe, Mikihito Takenaka

**Affiliations:** a Institute for Chemical Research, Kyoto University, Gokasho, Uji, 6110011, Japan; b Riken SPring-8 Center, 1-1-1 Kouto, Sayo, Hyogo 679-5148, Japan; c School of Computing, Tokyo Institute of Technology, 4259 Nagatsuta-cho, Midori-ku, Yokohama, Kanagawa 226-8503, Japan; d Department of Macromolecular Science and Engineering, Kyoto Institute of Technology, Kyoto 606-8585, Japan; e Department of Organic Materials Science, Yamagata University, Yonezawa, Yamagata 992-8510, Japan; f Japan Synchrotron Radiation Research Institute, 1-1-1 Kouto, Sayo, Hyogo 679-5198, Japan

**Keywords:** small angle X-ray scattering, computed tomography, SAXS-CT, diffuse scattering, soft-matter, image processing

## Abstract

Small-angle X-ray scattering (SAXS) coupled with computed tomography (CT), denoted SAXS-CT, has enabled the spatial distribution of the characteristic parameters (*e.g.* size, shape, surface, length) of nanoscale structures inside samples to be visualized. In this work, a new scheme with Tikhonov regularization was developed to remove the effects of artifacts caused by streak scattering originating from the reflection of the incident beam in the contour regions of the sample. The noise due to streak scattering was successfully removed from the sinogram image and hence the CT image could be reconstructed free from artifacts in the contour regions.

## Abstract 2

Small-angle X-ray scattering (SAXS) coupled with computed tomography (CT), denoted SAXS-CT, enables the spatial distribution of the characteristic parameters of nanoscale structures inside samples to be visualized. In this work, a new scheme with Tikhonov regularization was developed to remove the effects of artifacts caused by streak scattering originating from contour regions of the sample.

## 1. Introduction

Small-angle X-ray scattering (SAXS) has been widely used to characterize nanoscale structures within soft materials (Roe, 2000 ▸; Narayanan, 2009 ▸; Sakamoto & Hashimoto, 1995 ▸; Perret & Ruland, 1970 ▸; Takenaka, 2013 ▸). Recently, SAXS was combined with computed tomography (CT) to develop a powerful technique, known as the SAXS-CT method, for visualizing the spatial distribution of nanoscale structures inside samples (Feldkamp *et al.*, 2009 ▸; Schaff *et al.*, 2015 ▸; Skjønsfjell *et al.*, 2016 ▸; Georgiadis *et al.*, 2015 ▸; Hémonnot & Köster, 2017 ▸; Hu *et al.*, 2017 ▸; Conceição *et al.*, 2020 ▸). In SAXS-CT, we reconstruct the image of the sample from the SAXS intensity, while the image obtained from conventional CT is reconstructed from the transmittance of X-rays within the sample. Thus, the characteristic parameters obtained from the scattered intensity can be used as a contrast factor to form CT images. For example, when we construct a CT image from the intensity of the peak position characterizing the long spacing between the lamellar structures of crystalline polymers, we can obtain the spatial distribution of the lamellar structures (Schroer *et al.*, 2006 ▸; Stribeck *et al.*, 2006 ▸, 2008 ▸). Moreover, if we use the orientation factor obtained from 2D SAXS patterns, we can visualize the spatial distribution of nano­structural orientations.

In the SAXS-CT method, the basic experimental geometry is the same as that of conventional SAXS. The sample is irradiated by the narrow incident beam (*x* direction). SAXS-CT also needs a ‘scan’ along the axis perpendicular to the incident beam (*y* direction), achieved by moving the sample or the incident beam. The scan is repeated while the sample is rotated around the axis perpendicular to both the *x* and the *y* directions. The rotation angle (ϕ) corresponds to the ‘projection angle’ in conventional CT geometry. Thus, we obtain the 2D SAXS patterns at *y* and ϕ.

In this experimental geometry, we often face the serious problem that the reflection of incident X-rays interferes with the 2D SAXS patterns near the sample edge, which can result in anomalous scattering (artifacts) and seriously damage the reconstructed CT image. Voids within samples also cause strong scattering (Nozue *et al.*, 2007 ▸; Yang *et al.*, 1997 ▸; Etrillard *et al.*, 2005 ▸; Grubb & Murthy, 2010 ▸; Tomisawa *et al.*, 2019 ▸) which interferes with the accuracy of the reconstructed CT image. We can avoid the appearance of artifacts in CT images by analyzing the intensities at *q* positions outside these streaks or by changing the measurement conditions, such as the camera length. However, these approaches can be applied in only a limited number of cases.

Therefore, we attempted to remove streak-derived intensities from the sinogram (ϕ − *y*) by implementing Tikhonov-L1 optimization. We treated the removal procedure of the streak-derived noise as a constrained convex optimization problem, where we imposed Tikhonov-type regularization on the sinogram to exploit its underlying smoothness and used L1 norm regularization to characterize the sparsity of the streak-derived noise. For a sample, we employed a crystalline polymer exhibiting an isotropic peak in the SAXS scattering pattern originating from lamellar structures. We found almost identical SAXS patterns at any ϕ and *y* value except at the edge of the sample. When the incident beam irradiated the edge region of the sample, strong streak scattering was observed in the 2D SAXS pattern. By using this framework, we succeeded in obtaining a sinogram with the noise eliminated. However, we were able to apply this framework only to the data in the *y* direction at specific ϕ positions containing noise while preserving the *y* direction data at other ϕ positions. By reconstructing a CT image from this sinogram, we assessed the possibility of evaluation in the contour areas.

## 2. Experimental

### 2.1. Sample

We used high-density polyethyl­ene (HDPE, Tosoh Corporation), a typical crystalline polymer, as the sample in this study. In the SAXS region, we can observe the long spacing in lamellar structures (Schultz *et al.*, 1978 ▸; Strobl & Schneider, 1980 ▸; Bartczak *et al.*, 1992 ▸; Albrecht & Strobl, 1995 ▸; Hughes *et al.*, 1997 ▸; Tashiro *et al.*, 1998 ▸; Kishimoto *et al.*, 2020 ▸). Isotropic lamellar structures can be obtained by isothermal crystallization after the onset of quenching from the melt state to the crystallization temperature; thus, we can observe the isotropic scattering pattern of the sample at each irradiation position. Details of the polymer properties and measurement samples are given in the supporting information. The measurement sample had a cuboid shape, 0.86 × 0.98 × 22.5 mm (*x* × *y* × *z*) in size.

### 2.2. SAXS measurements

**Figure 1:**
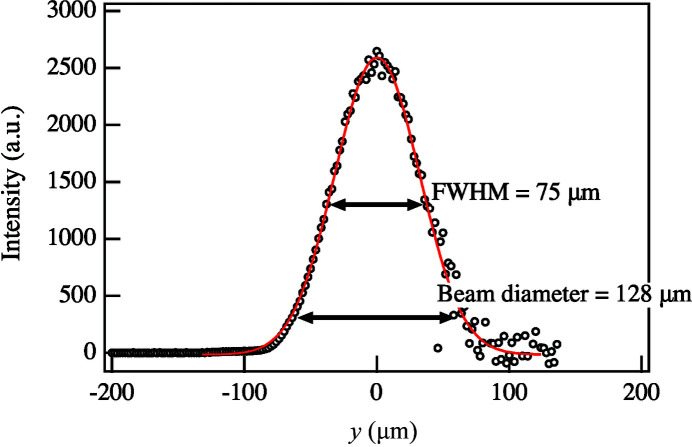
Horizontal beam profile measured at the sample position using blade scanning (black circles) and the fitted profile (red solid line).

SAXS measurements were performed at the second experimental hutch of beamline BL03XU at SPring-8, Frontier Soft-Material Beamline (FSBL), which is dedicated to SAXS experiments, using an intense beam (10^13^ photons s^−1^) with very low divergence [12.3 µrad (horizontal) × 1.1 µrad (vertical)]. The X-ray wavelength λ and the sample-to-detector distance were 0.1 nm and 4438 mm, respectively (Masunaga *et al.*, 2011 ▸). The beam size at the sample position was estimated using the method of scanning a blade. Fig. 1 ▸ shows the direct beam intensities when the blade is scanned horizontally at *y* = 2.0 µm. We fitted the beam profile with a Gaussian function, resulting in a full width at half-maximum (FWHM) of 75 µm. Furthermore, the beam diameter derived from the positions at which the intensity decayed from the peak intensity to 1/exp(2) was 128 µm. In the same manner, the beam diameter in the vertical direction was found to be 114 µm (see Fig. S1 of the supporting information). The scattering images were detected by a PILATUS 1M (Dectris Ltd.) with an exposure time of 0.5 s.

To reconstruct the images in the lateral directions using the CT method for the HDPE sample, we scanned a distance spanning 1.8 mm in steps of 30 µm in the direction perpendicular to the axis of rotation (*y* axis). The scanning step was approximately 1/4 of the incident beam width (the beam size in the horizontal direction). In the rotation scan ϕ, images were acquired in 3.0° steps for 0.0 ≤ ϕ < 180.0°.

### 2.3. Tikhonov-L1 minimization

This section is devoted to introducing our streak-removal framework based on Tikhonov-L1 minimization. Let **v** be a measured sinogram with noise. Then, our framework to eliminate noise with Tikhonov-L1 minimization is formulated as the following constrained optimization problem:

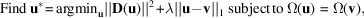

where u* is a sinogram with noise eliminated and **u** is the transient candidate of u*. The first term ∣∣**D**(**u**)∣∣^2^ is the Tikhonov regularization term for the image, where **D**(·) is an operator computing the 2D local differences of the input. ∣·∣^2^ denotes the squared norm (the sum of the squared values in the array), which plays a role in exploiting the inherent smoothness of the sinogram for estimation. The second term λ∣∣**u** − **v**∣∣_1_ is the L1 data fidelity with respect to the measured sinogram **v**, where ∣∣·∣∣_1_ denotes the L1 norm (the sum of the absolute values). The parameter λ balances these two terms. We characterized the noise by using the L1 norm, known as an effective sparsity-inducing criterion, since the noise (expressed as **u** − **v** in the optimization problem) is expected to be sparsely distributed on the measured sinogram.

The hard constraint Ω(**u**) = Ω(**v**) allowed us to maintain the measured values in **v** that reside in noise-free areas, where the operator Ω selects all the entries in those areas. If no information was known about the noise-free areas in advance, this constraint was simply removed.

In general, total variation (TV) type regularizations are better than the Tikhonov regularization for noise removal and compressed sensing of images with a piece-wise smooth structure (Chambolle, 2004 ▸; Bredies *et al.*, 2010 ▸). Our formulation is somewhat unusual in that it imposes hard constraints on the region specified by Ω, such that the value of the measurement **v** must be maintained. As a result, the optimization can only change the signal values in a small number of local regions, and since the local regions of the sinogram have a very smooth structure, the Tikhonov regularization, which is more strongly smoothing than TV-type regularization, is more appropriate.

Owing to the above mathematical characterizations, noise can be effectively removed while maintaining the detailed structure of the sinogram. To solve our optimization problem, we also developed an efficient algorithm based on a state-of-the-art optimization technique called the primal-dual splitting method (PDS; Condat, 2013 ▸).

We should note that the number of iterations required for convergence is generally larger in PDS than in the well known proximal algorithm of the alternating direction method of multipliers (ADMM; Boyd *et al.*, 2011 ▸). However, in ADMM, at each iteration, it is necessary to solve a quadratic minimization involving a matrix consisting of a difference operator **D** and a region selection operator Ω. As a result, it is necessary to solve a system of linear equations for each iteration, which incurs a considerable computational cost. On the other hand, PDS does not require such a step, and so PDS is less computationally expensive and easier to implement. For more information on proximal algorithms see Parikh & Boyd (2014 ▸) and Combettes & Pesquet (2011 ▸).

The programming language used was MATLAB. The optimization scheme iterated 2500 times, and the time to convergence was less than 1.0 s. The MATLAB code for the main part of the algorithm is shown in the supporting information.

## 3. Results and discussion

**Figure 2:**
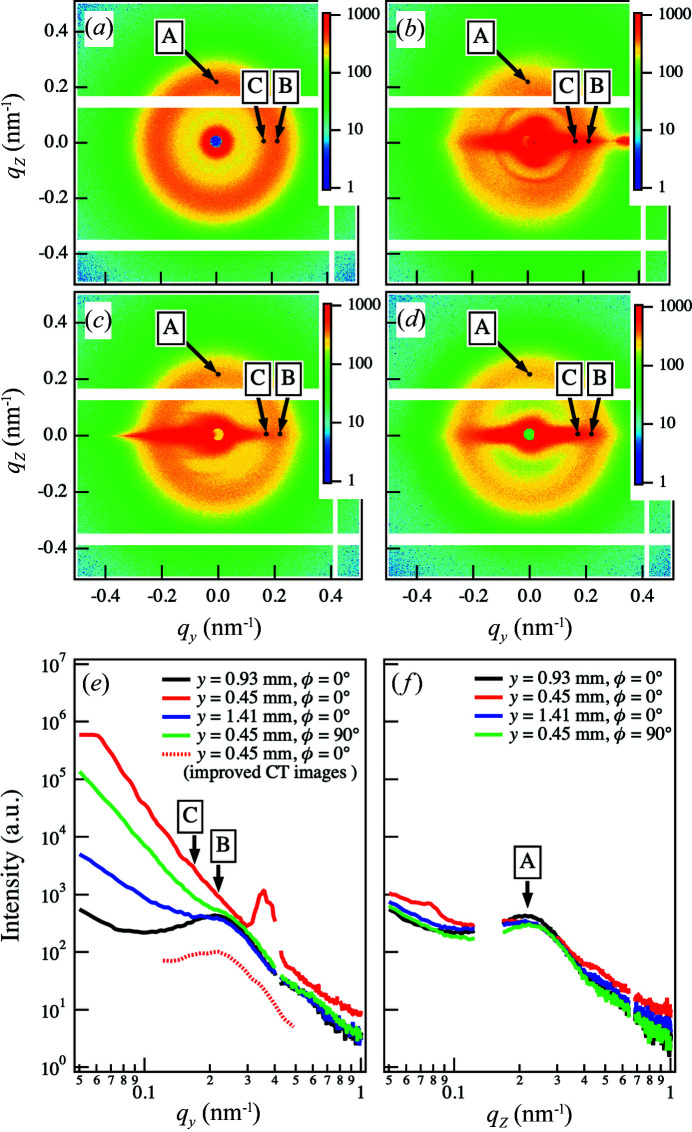
(*a*) 2D SAXS image without streak patterns. (*b*)–(*d*) Representative 2D SAXS images containing streak patterns. (*e*) 1D profile of each 2D SAXS image plotted along the horizontal direction. (*f*) 1D profile of each 2D SAXS image plotted along the vertical direction. A down-pointing arrow indicates the position at which the sinogram was obtained from the intensities. In (*e*), we added the 1D intensity profile obtained from the improved CT images.

Fig. 2 ▸(*a*) shows a 2D SAXS image acquired around the center part of the sample at ϕ = 0°. We observed a peak in the scattering pattern reflecting the periodicity of the lamellar structures. The peak intensity was isotropic, indicating that the orientation of the lamellar structures was isotropic. 1D profiles at various *y* values are plotted as a function of the *y* axis wavenumber *q_y_* in Fig. 2 ▸(*e*). We observed a broad peak at *q_y_* = 0.219 nm^−1^ [as indicated by A in Fig. 2 ▸(*f*)], which corresponds to the long spacing between lamellar structures. An isotropic pattern was observed when the incident X-rays were irradiated inside the sample. On the other hand, the scattering patterns at the edges were affected by the reflection of the incident beam. Figs. 2 ▸(*b*)–2(*d*) show representative 2D SAXS images observed when the incident X-rays irradiated the sample edges. Fig. 2 ▸(*b*) reveals strong streaks in the horizontal (*y* axis) direction from the beam center position; the streak is particularly strong at *q_y_* > 0 nm^−1^, indicating that reflection from one edge of the sample was observed without attenuation in this direction. Note that the peak intensities from 270 to 90° were higher than those from 90 to 270°. We observed these asymmetric intensities when the edge of the sample was irradiated. This asymmetry was due to the effects of air scattering before the sample. When irradiating the right edge of the sample, air scattering in the range 90–270° is attenuated by the sample. On the other hand, the air scattering at 270–90° does not pass through the sample. As a result, the left and right halves of the image might show different intensities. Fig. 2 ▸(*b*) shows that the diffraction peak appeared at around *q* = 0.138 nm^−1^. The reflected beam from the edge of the sample is intense. We attributed this diffraction to the reflected light irradiating the Kapton (the window material of the vacuum path). In Fig. 2 ▸(*c*), a strong streak was observed at *q_y_* < 0 nm^−1^ at the sample edge on the other side. We also observed streaks in the horizontal direction in the 2D SAXS image at ϕ = 90° [Fig. 2 ▸(*d*)]. As shown in Fig. 2 ▸(*e*), at *y* = 0.45 mm and ϕ = 0° [corresponding to Fig. 2 ▸(*b*)], the streak intensity overwhelmed the peak intensity of lamellar structures in all *q_y_* regions. Consequently, we were not able to identify the scattering peak [as indicated by B in Fig. 2 ▸(*e*)]. At *y* = 1.41 mm and ϕ = 0° [corresponding to Fig. 2 ▸(*c*)], a high streak intensity was observed at *q_y_* < 0.195 nm^−1^, but it did not affect the peak profile. For *q_y_* ≳ 0.195 nm^−1^, the intensity was almost identical to that obtained from Fig. 2 ▸(*a*). The scattering profile at *y* = 0.45 mm and ϕ = 90° in Fig. 2 ▸(*e*) was affected by the streak intensity, although the peak was observed in the profile. These results indicate that the intensity at the peak position *q_y_* = 0.219 nm^−1^ was affected by the streak intensity. The effect of the streak intensity became increasingly significant with decreasing *q_y_* at the sample edges. The disturbance caused by the streak scattering can be discerned at position C.

Note that the streak scattering from the edges in this case did not affect the peak intensity parallel to the axis of rotation or *q*_*z*_ direction, as indicated by point A in Figs. 2 ▸(*a*)–2 ▸(*d*). The scattered intensities along *q*_*z*_ are plotted in Fig. 2 ▸(*f*). We could not detect any effects from the streak scattering at any value of *y*, indicating that the sinogram and the reconstructed image were not affected by the streak-scattering disturbance. Thus, we first reconstructed a CT image from the intensity at point A to obtain a sinogram free from streak-scattering noise.

**Figure 3:**
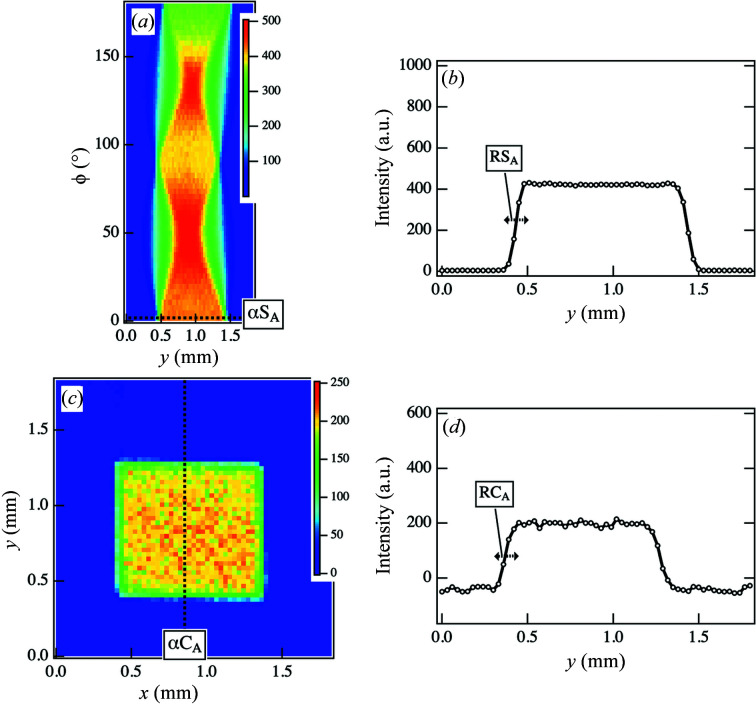
(*a*) Original sinogram obtained from the scattering intensities at point A in the 2D SAXS images at each ϕ and *y*. (*b*) Cross-sectional intensity profile at ϕ = 0.0° along α*S*_A_ in (*a*). (*c*) Original CT image reconstructed from (*a*). (*d*) Cross-sectional intensity profile at *x* = 0.84 mm along αC_A_ in (*c*).

Fig. 3 ▸(*a*) shows the sinogram obtained from the intensity at point A in the parameter space ϕ and *y*. The sinogram does not contain any abrupt intensity spikes. The intensity profiles for ϕ = 0° in Fig. 3 ▸(*a*) are plotted as a function of *y* (as indicated by the dotted line αS_A_) in Fig. 3 ▸(*b*). An inclination can be observed at both ends of the profile. This inclination originated from the change in the irradiation area of the incident beam at the edges of the samples, causing the width of the inclination *y* to correspond to the beam diameter. However, the width of the inclination at the edges of the sample (at ϕ = 0°) was estimated at 90 < *y* < 150 µm, which is consistent with the horizontal beam diameter [as indicated by RS_A_ in Fig. 3 ▸(*b*)]. The contour areas corresponding to the inclination can be observed in the CT image reconstructed from the sinogram, as shown in Fig. 3 ▸(*c*). Fig. 3 ▸(*d*) shows the cross-sectional intensity profile for *x* = 0.84 mm as a function of *y* [as indicated by the dotted line αC_A_ in Fig. 3 ▸(*c*)]. The inclination of the contour area was again evaluated at 90 < *y* < 150 µm [as indicated by RC_A_ in Fig. 3 ▸(*d*)], which is consistent with the values estimated in the sinogram. Thus, when streak scattering does not affect the reconstruction, inclination of the beam width can be observed at the edge of the sample in the CT image.

**Figure 4:**
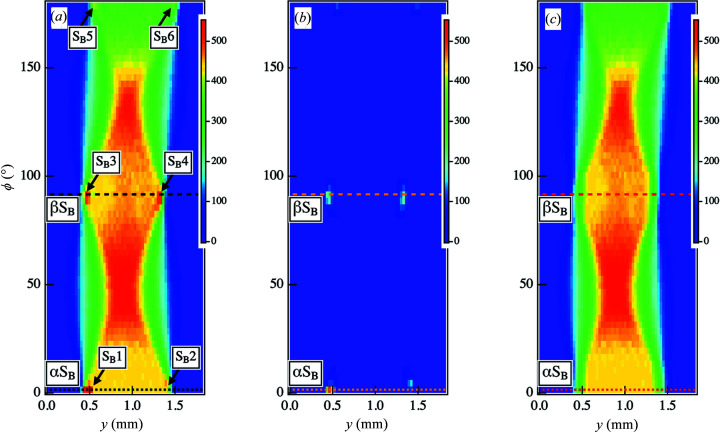
(*a*) Original sinogram obtained from the scattering intensities at point B in the 2D SAXS images at each ϕ and *y*. The positions of the arrows indicate where the streak intensity was superimposed on the scattering intensity. (*b*) Sinogram of the noise components removed using our framework of Tikhonov regularization. (*c*) Sinogram of the signal components obtained after noise elimination.

**Figure 5:**
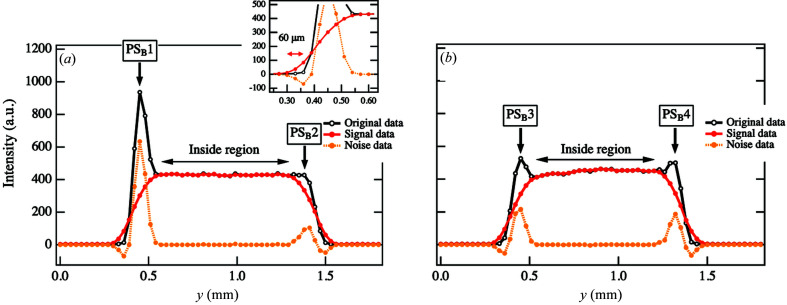
(*a*) Cross-sectional intensity profiles at ϕ = 0.0° along αS_B_ in Figs. 4 ▸(*a*)–(*c*). (*b*) Cross-sectional intensity profiles at ϕ = 90.0° along βS_B_ in Figs. 4 ▸(*a*)–(*c*).

Next, we will discuss how streak scattering affects CT images. We obtained sinograms from the scattering intensities at various *q*_y_ positions. Fig. 4 ▸ shows the sinograms of the scattered intensity at a given *q_y_* = 0.219 nm^−1^ corresponding to the peak position in the parameter space of ϕ and *y*. We observed spots at the *y* positions along the sample edges at approximately ϕ = 0, 90 and 180°, as indicated by S_B_1 − S_B_6 in Fig. 4 ▸(*a*). The intensity profiles along the dotted line αS_B_ at ϕ = 0° in Fig. 4 ▸(*a*) are plotted as a function of *y* in Fig. 5 ▸(*a*). A distinct peak in the profile was found at *y* = 0.45 mm [as indicated by PS_B_1 in Fig. 5 ▸(*a*)]. The peak width was evaluated to be 120 < *y* < 180 µm, consistent with the horizontal beam diameter. This agreement indicates that streak scattering appears once the incident beam irradiates the edge of the sample at *y* = 0.45 mm. On the other hand, we were not able to observe a distinct peak at another edge of the sample, *y* = 1.38 mm [as indicated by PS_B_2 in Fig. 5 ▸(*a*)]. However, the width of the inclination was in the range 60 < *y* < 120 µm, which is approximately 47–94% smaller than the horizontal beam diameter. This decrease in the width originated from the fact that streak scattering compensates for the gradual decrease in the intensity at the inclination area. Although the streak scattering at the edge of *y* = 1.38 mm did not appear as strong, the profile was modified by streak scattering.

The intensity profile along the dotted line βS_B_ at ϕ = 90° in Fig. 4 ▸(*a*) is shown in Fig. 5 ▸(*b*). We discerned peaks around the sample edge positions of *y* = 0.45 mm and *y* = 1.32 mm [as indicated by PS_B_3 and PS_B_4 in Fig. 5 ▸(*b*)], and the peak widths were estimated to be 120 < *y* < 180 µm and 90 < *y* < 150 µm, respectively. The horizontal beam diameters were found to be within these ranges.

**Figure 6:**
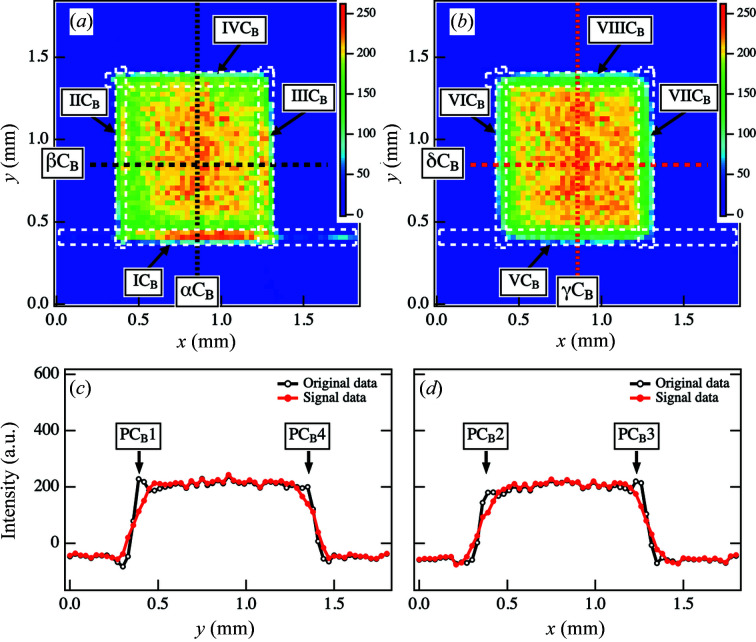
(*a*) Original CT image reconstructed from the original sinogram in Fig. 4 ▸(*a*). (*b*) Improved CT image reconstructed from the sinogram of the signal components in Fig. 4 ▸(*c*). (*c*) Cross-sectional intensity profiles at *x* = 0.84 mm along αC_B_ in (*a*) and γC_B_ in (*b*). (*d*) Cross-sectional intensity profiles at *y* = 0.84 mm along βC_B_ in (*a*) and δC_B_ in (*b*).

The CT image reconstructed from the original sinogram of Fig. 4 ▸(*a*) is shown in Fig. 6 ▸(*a*). Artifacts are apparent in the *x* direction at the IC_B_ region in Fig. 6 ▸(*a*) and are attributed to the intensities in parts S_B_1 and S_B_5 in Fig. 4 ▸(*a*). Additionally, we observed partial artifacts in regions IIC_B_ and IIIC_B_ in the CT image due to the intensities in the S_B_3 and S_B_4 parts of Fig. 4 ▸(*a*). In contrast, there were no artifacts clearly detected in region IVC_B_ in the CT image.

We applied our framework to the sinogram in Fig. 4 ▸(*a*) and successfully extracted the abrupt increase within the sinogram as noise, as shown in Fig. 4 ▸(*b*). Then, we subtracted the noise from the sinogram in Fig. 4 ▸(*a*) and obtained the sinogram of the signal components [Fig. 4 ▸(*c*)]. In this case, we implemented Tikhonov regularization while specifying only the profiles in the *y* direction at the ϕ positions ϕ = 0–9°, ϕ = 81–96° and ϕ = 174–177°. In contrast, the original data in the *y* direction at the other ϕ positions were preserved. The intensity profiles for ϕ = 0° in Figs. 4 ▸(*b*) and 4(*c*) [as indicated by the dotted lines αS_B_ in Figs. 4 ▸(*b*) and 4(*c*)] are plotted in Fig. 5 ▸(*a*). In the profile of the noise component, the peak was identified at *y* = 0.45 mm [as indicated by PS_B_1 in Fig. 5 ▸(*a*)]. Additionally, we confirmed the peak on the opposite side at approximately *y* = 1.41 mm (PS_B_2), although it was difficult to determine the peak in the original profile. The profile from the signal component is characterized by smooth intensity decays in these regions. However, owing to the smooth intensity decay in the tail areas, the inclination widths in both regions were approximately 60 µm larger than those obtained from the original sinogram. In addition to the rapid increase in peak intensity values, the true signal data in this region also changed significantly; as a consequence, smoothing resulted in a broader intensity decay in the tail. Because of the absence of the removed noise for 0.57 ≤ *y* ≤ 1.29 mm, the intensities were the same as the original intensities. As shown in Fig. 5 ▸(*b*), we could also remove the peaks at the edges or *y* = 0.45 mm and *y* = 1.32 mm for ϕ = 90° [as indicated by PS_B_3 and PS_B_4 in Fig. 5 ▸(*b*)].

Fig. 6 ▸(*b*) shows the CT image reconstructed from the signal-component sinogram in Fig. 4 ▸(*c*). Comparison with the original CT image reconstructed from the original sinogram shows that the artifacts in regions IC_B_, IIC_B_ and IIIC_B_ are missing from the improved CT image. Fig. 6 ▸(*c*) shows the cross-sectional intensity profiles for *x* = 0.84 mm in Figs. 6 ▸(*a*) and 6(*b*) as a function of *y* [as indicated by the dotted lines αC_B_ in Fig. 6 ▸(*a*) and γC_B_ in Fig. 6 ▸(*b*)]. In Fig. 6 ▸(*d*), we show the cross-sectional intensity profiles for *y* = 0.84 mm in Figs. 6 ▸(*a*) and 6(*b*) as a function of *x* [as indicated by the dotted lines βC_B_ in Figs. 6 ▸(*a*) and δC_B_ in Fig. 6 ▸(*b*)]. The removal of artifacts resulted in improved profiles at approximately *y* = 0.39 mm [as indicated by PC_B_1 in Fig. 6 ▸(*c*)] and approximately *x* = 0.39 mm and *x* = 1.26 mm [as indicated by PC_B_2 and PC_B_3 in Fig. 6 ▸(*d*)]. Additionally, the profile at the sample edge position *y* = 1.35 mm was improved [as indicated by PC_B_4 in Fig. 6 ▸(*c*)].

**Figure 7:**
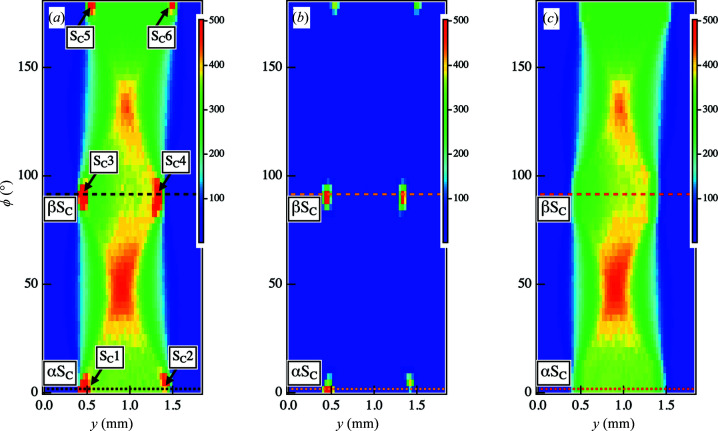
(*a*) Original sinogram obtained from the scattering intensities at point C in the 2D SAXS images at each ϕ and *y*. The positions of the arrows indicate where the streak intensity was superimposed on the scattering intensity. (*b*) Sinogram of the removed noise components. (*c*) Sinogram of the signal components obtained after noise elimination.

**Figure 8:**
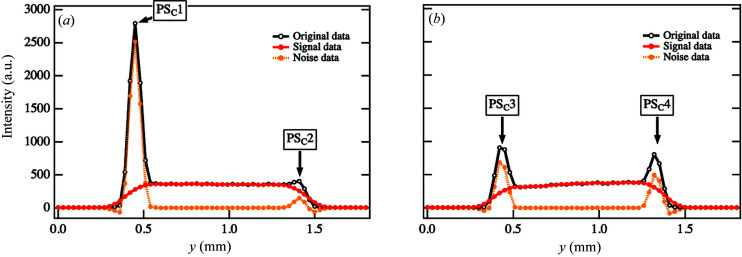
(*a*) Cross-sectional intensity profiles at ϕ = 0.0° along αS_C_ in Figs. 7 ▸(*a*)–(*c*). (*b*) Cross-sectional intensity profiles at ϕ = 90.0° along βS_C_ in Figs. 7 ▸(*a*)–(*c*).

Figs. 4 ▸–6 ▸ ▸ shows an attempt to remove the noise for a signal-to-noise intensity ratio of about 200%. We examined the upper limit of the signal-to-noise ratio of the framework using these parameter values. As a result, we were able to remove noise with an intensity ratio of up to 2000%, as shown in Figs. 7 ▸–9 ▸ ▸. We have shown the results of applying our framework to the sinogram obtained from the scattering intensities in the lower *q_y_* region [as indicated by C in Figs. 2 ▸(*a*)–2(*d*)]. As shown in the 1D profiles, the effect of streak scattering became more evident with decreasing *q_y_*. As shown in Fig. 2 ▸(*e*), the differences between the profile at *y* = 0.93 mm and ϕ = 0° (black solid line), and that at *y* = 0.45 mm and ϕ = 0° (red solid line) increased with decreasing *q_y_*. Thus, the sinogram obtained from the intensity at *q_y_* = 0.170 nm^−1^ in Fig. 7 ▸(*a*) exhibited more pronounced spots than that at *q_y_* = 0.219 nm^−1^ in Fig. 7 ▸(*a*) [as indicated by S_C_1–S_C_6 in Fig. 7 ▸(*a*)]. The intensity profile for ϕ = 0° (αS_C_) is plotted as a function of *y* in Fig. 8 ▸(*a*). We observed peaks at both edges of the sample at *y* = 0.45 mm and *y* = 1.41 mm [as indicated by PS_C_1 and PS_C_2 in Fig. 8 ▸(*a*)]. The widths of both peaks were estimated to be 120 < *y* < 180 µm, consistent with the horizontal beam diameter. We then applied Tikhonov regularization to the sinogram to remove the noise under the same angular range conditions (ϕ) specified in Fig. 4 ▸(*a*). We were able to extract the noise components successfully, as shown in Fig. 7 ▸(*b*), and an improved sinogram was obtained by subtracting the noise component from the original image, as shown in Fig. 7 ▸(*c*). The intensity profiles demonstrate that the noise components were only the peak profiles from the edge areas [as indicated by PS_C_1 and PS_C_2 in Fig. 8 ▸(*a*) and PS_C_3 and PS_C_4 in Fig. 8 ▸(*b*)]. On the other hand, the intensity profiles from the signal component in these areas became smoother due to the removal of noise.

**Figure 9:**
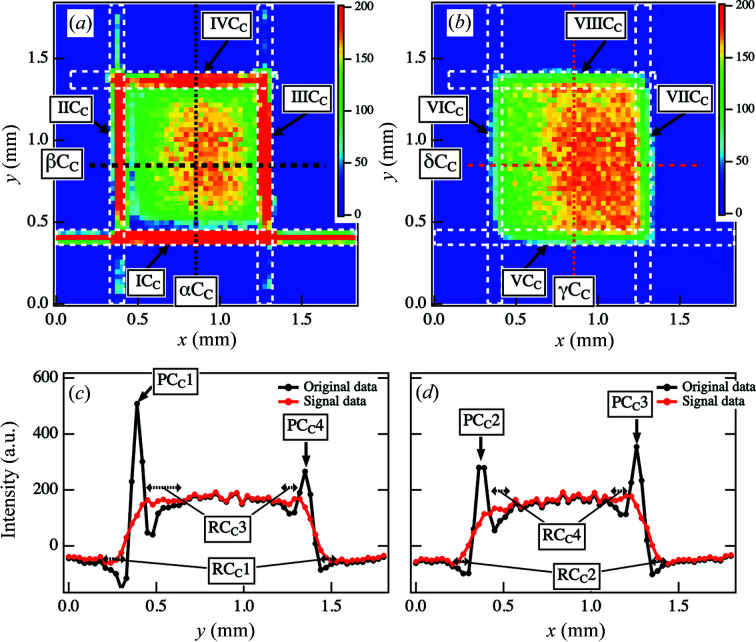
(*a*) Original CT images reconstructed from the original sinogram in Fig. 7 ▸(*a*). (*b*) Improved CT images reconstructed from the sinogram of the signal components in Fig. 7 ▸(*b*). (*c*) Cross-sectional intensity profiles at *x* = 0.84 mm along α*C*_C_ in (*a*) and γ*C*_C_ in (*b*). (*d*) Cross-sectional intensity profiles at *y* = 0.84 mm along β*C*_C_ in (*a*) and δ*C*_C_ in (*b*).

Fig. 9 ▸(*a*) shows the CT image reconstructed from the original sinogram. Streak scattering induced artifacts not only in the contour areas [as indicated by IC_C_, IIC_C_, IIIC_C_ and IVC_C_ in Fig. 9 ▸(*a*)] but also outside the sample. Since the relative spot intensities at the six positions in the sinogram increased, the artifacts in the *x* and *y* directions in the original CT image became more pronounced. Furthermore, when the spot intensities in the sinogram increased in the ϕ directions, artifacts were also observed for the corresponding ϕ directions. These artifacts overlapped, resulting in a broader width in the *x* and *y* directions. The cross-sectional intensity profile at *x* = 0.84 mm [as indicated by the dotted lines αC_C_ in Fig. 9 ▸(*a*) and γC_C_ in Fig. 9 ▸(*b*)] is plotted as a function of *y* in Fig. 9 ▸(*c*), and the cross-sectional intensity profile at *y* = 0.84 mm is plotted as a function of *x* in Fig. 9 ▸(*d*) [as indicated by the dotted lines βC_C_ in Fig. 9 ▸(*a*) and δC_C_ in Fig. 9 ▸(*b*)]. Strong artifacts are visible as broad peaks at approximately *y* = 0.39 mm and *y* = 1.35 mm [as indicated by PC_C_1 and PC_C_4 in Fig. 9 ▸(*c*)] and at approximately *x* = 0.39 mm and *x* = 1.26 mm [as indicated by PC_C_2 and PC_C_3 in Fig. 9 ▸(*d*)]. In both profiles, the peak profiles prevented us from identifying the edges of the sample. Moreover, an oscillation behavior was observed around the peaks [as indicated by RC_C_1 and RC_C_3 in Fig. 9 ▸(*c*) and RC_C_2 and RC_C_4 in Fig. 9 ▸(*d*)]. In ordinary CT reconstruction calculations, the sinogram is subject to filtering and then backprojection. Since the filtering process is mathematically identical to differentiation, impact-type noise such as streaks can cause unexpectedly large errors. Furthermore, due to the resolution limit of numerical differentiation, damping waves propagate around the impacts, as seen in regions RC_C_1 and RC_C_3 in Fig. 9 ▸(*c*) and RC_C_2 and RC_C_4 in Fig. 9 ▸(*d*). These factors generated false signals in the projection direction, yielding the streak lines in the rectangular dotted boxes in the reconstructed images. Thus, we were not able to evaluate a large area in the reconstructed CT image from the sinogram affected by strong streak scattering.

Fig. 9 ▸(*b*) shows the reconstructed CT image from the sinogram of the signal component. As a result of removing the artifacts, we were able to observe the contour areas [as indicated by VC_C_, VIC_C_, VIIC_C_ and VIIIC_C_ in Fig. 9 ▸(*b*)]. As shown in Figs. 9 ▸(*c*) and 9(*d*), the cross-sectional intensity profiles became smooth after applying our framework. The profiles also reveal improved intensities in the background region. Moreover, the intensity values decreased more effectively in the interior regions, which allowed us to obtain information on the spatial distribution of the structures therein [as indicated by RC_C_3 and RC_C_4 in Figs. 9 ▸(*c*) and 9 ▸(*d*)]. Although only the ranges 0.66 < *y* < 1.20 mm and 0.54 < *x* < 1.08 mm inside the sample could be evaluated in the original CT image, we could evaluate the ranges 0.42 < *y* < 1.35 mm and 0.39 < *x* < 1.26 mm in the improved CT image. Furthermore, estimating the inclination width of the sample edge at position PC_C_1 was difficult in the original CT image because of the large propagation effect of the attenuated wave described above, but in the improved CT image, the width at the same edge could be estimated as 150 < *y* < 210 µm [Fig. 9 ▸(*c*)]. This width was approximately 60 µm larger than the value estimated at the same edge of the sample in the CT image in Fig. 3 ▸(*d*). The reason for this increased inclination width was its increase along the edge at ϕ = 0° in the sinogram after the removal of noise.

These results show that our framework is applicable even for the removal of high noise intensities. We expect that this framework will also be useful in removing the high-intensity components that partially occurred inside the sample.

Finally, we compare the scattering intensity profile obtained from the improved CT image with the original. We also improved the CT images at each position from *q_y_* = 0.12 nm^−1^ to *q_y_* = 0.49 nm^−1^ in addition to the B and C positions. The averaged intensity along the *x* direction at the edge position *y* = 0.45 mm in these images is plotted against each *q_y_* as shown in Fig. 2 ▸(*e*) (red dashed line). On the lower *q_y_* side, this is the upper limit of the signal-to-noise ratio of this framework based on the set parameter values. On the other hand, at the higher *q*_*y*_ side, the framework is applied up to the *q_y_* region where streaks affect the signal intensities. As a result, we plotted the intensity profile in the limited *q_y_* region. In contrast to the case for the original profile with *y* = 0.45 mm and ϕ = 0°, we could identify the peak of the lamellar structure at *q*_*y*_ = 0.219 nm^−1^, suggesting that the method can remove the effect of streak scattering from the measured scattering function. Our method can effectively remove noise without losing any information in the sinogram, provided that the dimension of the measurements is less than half of the dimension **v**, due to the powerful smoothing capability of the Tikhonov regularization. We suggest that this framework could be used in applications other than streaks coming from the sample edge, such as those originating from voids in the interior.

## 4. Conclusions

We have developed a framework with Tikhonov regularization to remove the effects of artifacts originating from reflections that occur at the edges or the voids of a sample during SAXS-CT. We applied the framework to the reconstruction of a CT image for the spatial distribution of the lamellar structure of an HDPE sample. The SAXS data used for the reconstruction of the CT image were affected by the streak scattering originating from the reflections at the edges of the sample. We were able to remove the effect of streak scattering on the reconstruction of the CT image by employing the proposed framework and obtained an improved CT image, whereas disturbances were found in the CT image obtained without the framework. This technique is useful for removing spot-like noise not only for SAXS-CT but also for conventional X-ray CT.

## Supplementary Material

Supporting table, figure and code. DOI: 10.1107/S1600576721011523/ge5111sup1.pdf
