# Supplementary material for: Artifact removal in the contour areas of SAXS-CT images by Tikhonov-L1 minimization
Source: J Appl Crystallogr. 2021 Nov 30;54(Pt 6):1784–92. doi: 10.1107/S1600576721011523 (PMC8662970; doi:10.1107/S1600576721011523)
Supplement: Supplementary file 1 [file j-54-01784-sup1.pdf]

JOURNAL OF APPLIED CRYSTALLOGRAPHY

Volume 55 (2022)

Supporting information for article:

Artifact removal in the contour areas of SAXS-CT images by Tikhonov-L1 minimization

Hiroki Ogawa, Shunsuke Ono, Yuki Watanabe, Yukihiro Nishikawa, Shotaro Nishitsuji, Taizo Kabe and Mikihito Takenaka

Polymer properties and measurement samples

The properties of the high-density polyethylene (HDPE, Tosoh Corporation) are shown below.

M_n (the number average molecular weight)	56,700
M_w (the weight average molecular weight)	12,900
Polydispersity index	4.4
MFR (melt mass-flow rate)	20 g/10min
Density	0.955 g/cm^3

The HDPE pellets were first melted at 220°C for 15 minutes using a hot press, after which heat compression molding was applied at 220°C for 15 minutes at 15 MPa, and then they were cooled to room temperature by water to obtain flat plates of approximately 2× 75 × 50 mm^3. The obtained flat plates were then cut on four sides into a cuboid with dimensions of 0.86 × 0.98 × 22.5 mm^3 at -30°C using a microtome to make measurement samples.

Vertical beam profile at the sample position

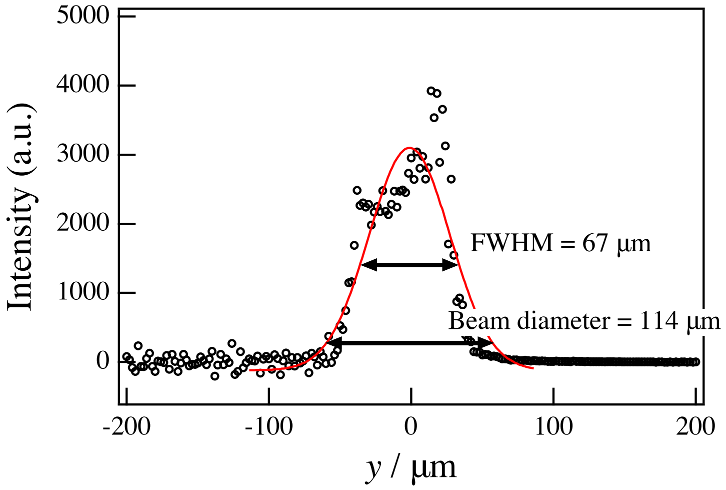

Figure S1. Measured vertical beam profile at the sample position using blade scanning (black circles) and the fitted profile (red solid line).

MATLAB Implementation code of our main part

```
for i = 1:maxIter  upre = u;

  % update u

  u = u - gamma1*(Dt(z1) + z2);

  utemp = v;

  utemp(nInd) = u(nInd);

  u = utemp;  % update z1

  z1 = z1 + gamma2*D(2*u - upre);

  temp1 = z1/gamma2;

  temp1 = temp1/(2/gamma2 + 1);

  z1 = z1 - gamma2*temp1;  % update z2

  z2 = z2 + gamma2*(2*u - upre);

  temp2 = z2/gamma2;

  temp2v = temp2-v;

  temp2 = v + sign(temp2v).*max(abs(temp2v)-lambda/gamma2, 0);

  z2 = z2 - gamma2*temp2;  % stopping condition

  res = u - upre;

  error = norm(res(:),2)/norm(u(:),2);

  if i>10 && error < stopcri

    break;

  end
```
